# Synthesis, Electrochemical and Fluorescence Properties of the First Fluorescent Member of the Ferrocifen Family and of Its Oxidized Derivatives

**DOI:** 10.3390/molecules27196690

**Published:** 2022-10-08

**Authors:** Charles Fayolle, Pascal Pigeon, Nathalie Fischer-Durand, Michèle Salmain, Olivier Buriez, Anne Vessières, Eric Labbé

**Affiliations:** 1 PASTEUR, Département de chimie, École Normale Supérieure, PSL University, Sorbonne Université CNRS, 75005 Paris, France; 2 Institut Parisien de Chimie Moléculaire (IPCM), CNRS, Sorbonne Université, 75005 Paris, France; 3 ENSCP Chimie ParisTech, PSL University, 75005 Paris, France

**Keywords:** ferrocene, coumarin, fluorescence switching, PET, redox state

## Abstract

The first fluorescent ferrociphenol derivative (P797) has been synthesized via McMurry cross-coupling followed by copper-catalyzed [3 + 2] azide-alkyne cycloaddition of the fluorescent group coumarin. Cyclic voltammograms of P797 exhibit either a monoelectronic oxidation wave ascribed to the ferrocene Fe(II) → Fe(III) conversion or a three-electron oxidation process in the presence of a base, leading to a Fe(III) quinone methide adduct. This general sequence is consistent with those previously described for non-fluorescent ferrociphenols. Furthermore, the fluorescence properties of P797 and its oxidized intermediates appear to strongly depend on the redox state of the ferrocene group. Indeed, electrochemical generation of Fe(III) (ferrocenium) states markedly increases the fluorescence emission intensity. In contrast, the emission of the Fe(II) (ferrocene) states is partially quenched by photoinduced electron transfer (PET) from the Fe(II) donor to the coumarin acceptor and by concentration-dependent self-quenching. Owing to its switchable fluorescence properties, complex P797 could represent an innovative and useful tool to study the biodistribution and the redox state of ferrocifens in cancer cells.

## 1. Introduction

Fluorescence optical imaging is a broadly used technique for monitoring and tracking molecules which has found applications in drug delivery [1] and in vivo tumor imaging [2] to name a few. Fluorescent probes based on organic dyes such as BODIPY [3,4] or polymers [5] have become instrumental in biology to visualize a large variety of (bio)molecules [6,7] and metal ions [8]. The capacity to associate a fluorescent probe to a specific redox state is also very interesting, as fluorescence emission can be switched on and off as a function of the oxidizing environment [9]. For instance, molecules featuring this property are extremely useful for studying the metabolism of cells in which reactive oxygen and nitrogen species (ROS and RNS) are overproduced [10].

Fluorescent probes comprising a redox-active ferrocene entity are particularly attractive because of (i) the reversible redox change between the Fe(II) and Fe(III) states that occurs at biologically meaningful potential and (ii) the known ability of ferrocene to quench fluorescence [11]. Intramolecular fluorescence extinction generally involves a photoinduced electron transfer (PET) mechanism where electron transfer proceeds from a donor group (here ferrocene) to the fluorophore excited state acting as an acceptor [12]. Furthermore, PET is aborted upon oxidation of ferrocene to its ferricenium form, resulting in fluorescence emission enhancement. In this respect, several ferrocene–naphthoquinone dyads were designed to study the factors influencing the ET process [13]. In addition, ferrocene–naphthalimide–piperazine triads have been shown to allow simultaneous pH and redox sensing [14]. Recently, Thakur et al. successfully designed ferrocene–coumarin platforms as PET-based chemosensors of Fe^3+^ and Cu^2+^ cations [15,16]. In the field of anticancer metallodrugs, Mokhir et al. have introduced several ROS-sensitive, ferrocene–fluorophore conjugates whose fluorescence emission is partially quenched by PET, whereas their oxidation results in an increased fluorescence emission either in vitro or in cancer cells due to PET release [17,18,19]. 

Ferrocifens, i.e., complexes connecting a tamoxifen skeleton to a ferrocenyl group, have been extensively explored in the last two decades for their potent anticancer properties [20]. Their metabolism in oxidative environments such as those met in cancer cells relies on a complex oxidation sequence that has already been explored for several members of this family, essentially ferrociphenols [21,22], ferrocenyl anilines [23,24], and ferrocenophanic suberamides [25]. Characterization of the corresponding intermediates and their lifetimes owes much to the concepts developed by Anny Jutand and Christian Amatore on the electrochemical determination of mechanisms met in organometallic chemistry [26,27]. Christian Amatore’s contribution to the electrochemistry study of ferrocifentype organometallics is seminal [21] and enables the characterization of their oxidative pathways. The work presented here is a tribute to his major contributions in this field.

**Chart 1:**
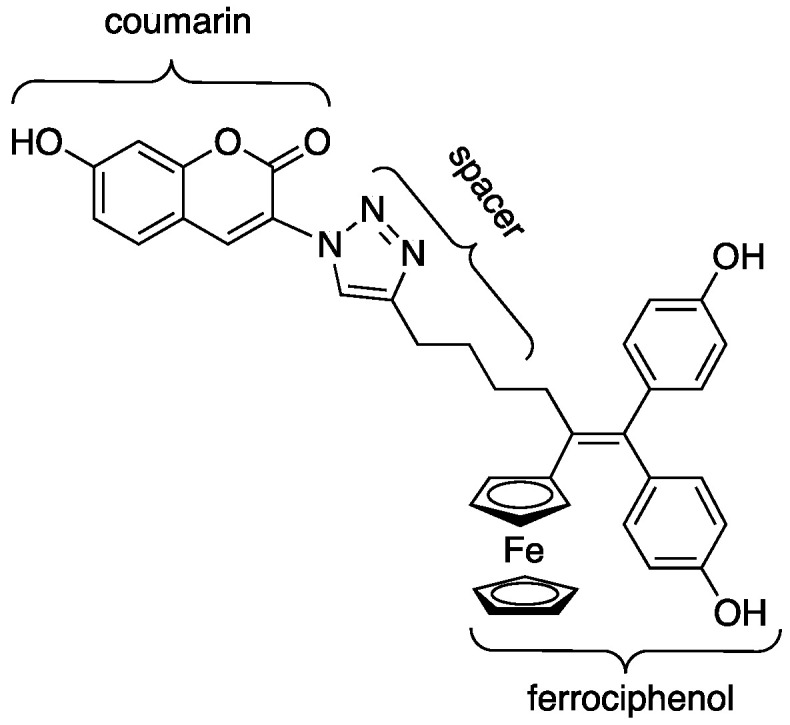
Chemical structure of P797.

Some of us recently uncovered the redox switchable properties of ferrocene–rhodamine dyads [28], as well as the selective electrochemical bleaching of the fluorescence emitted by NBD-tagged lipids and peptides [29,30] (NBD = 7-nitrobenz-2-oxa-1,3-diazole). Here, we report the synthesis of P797 (Chart 1), the first fluorescent member of the ferrocifen family for which a coumarin fluorophore was connected to the ferrociphenol skeleton via a spacer arm of 4-carbon alkyl chain (Chart 1). The electrochemical and fluorescent properties of P797 in the Fe(II) and Fe(III) states were explored, enabling to unambiguously establish its oxidation sequence in the absence or presence of a base.

## 2. Results and Discussion

### 2.1. Synthesis of Ferrociphenol Coumarin Dyads

**Scheme 1:**
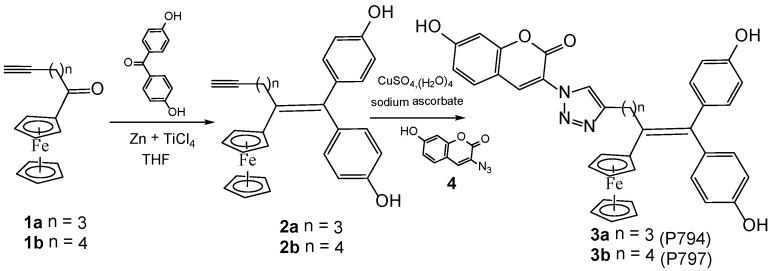
Synthesis of ferrociphenol coumarin dyads P794 and P797.

McMurry coupling between the alkynyl ketone **1b** and 4,4′-dihydroxybenzophenone afforded the alkynyl ferrociphenol **2b** in 27% yield. Complex P797 was finally obtained in 68% yield by copper-catalyzed [3 + 2] azide-alkyne cycloaddition between **2b** and 3-azido-7-hydroxycoumarin **4** (Scheme 1). P794, a complex with a shorter three-carbon alkyl chain was also prepared by the same reaction sequence. Attempts to synthesize a derivative with a two-carbon spacer arm were unsuccessful because of rapid decomposition of the alkynyl intermediate.

### 2.2. Fluorescence Properties of P794 and P797

**Figure 1:**
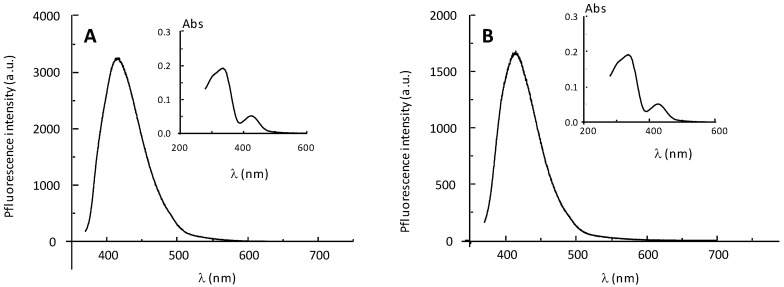
UV-vis absorption (insets) and fluorescence emission spectra of P797 ((**A**) left graph) and P794 ((**B**) right graph) at 1 × 10^−5^ mol.dm^−3^ in acetonitrile + TBABF_4_ at 0.1 mol.dm^−3^. Excitation wavelength (λ_ex_) = 350 nm for fluorescence spectra.

UV-visible and fluorescence spectra of the two newly synthesized complexes P794 and P797 are displayed in Figure 1. 

As shown in the inset, the two complexes have similar UV-vis spectra, with maximum absorption at 337 and 425 nm. For the same excitation wavelength (350 nm), the fluorescence intensity of P797 is twice that of P794. This significant difference in fluorescence emission most likely arises from a quenching process which is more efficient in P794. As discussed below, fluorescence is affected by a photoinduced electron transfer (PET) from the Fe(II) to the coumarin, a mechanism which rate and efficiency are known to depend on the structure and distance between the donor (Fe(II) in our work) and the acceptor (coumarin). Electron transfers are fundamental in redox chemistry and photochemistry and their rates depend on the reaction coordinate between the donor and the acceptor [31], a feature which has been illustrated using self-assembled monolayers (SAMs) of various chain length separating a porphyrin photosensitizer and a ferrocene group [32]. Since P797 exhibits a higher fluorescence intensity, we decided to carry out the following experiments with P797 only.

### 2.3. Electrochemical Behavior of P797 in Acetonitrile in the Absence and Presence of Imidazole Used as a Base

**Figure 2:**
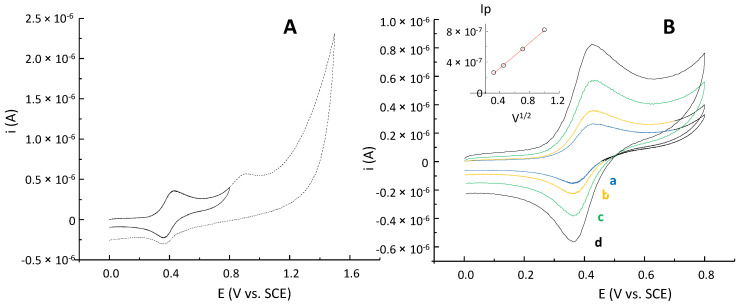
Cyclic voltammograms (CVs) of P797 (1 mmol.dm^−3^). (**A**) CVs of P797 in acetonitrile (solid line); in acetonitrile + TBABF_4_ 0.1 mol.dm^−3^ (dotted line) at a Pt disk electrode (diam 0.5 mm). Scan rate: 0.1 V.s^−1^; (**B**) CVs of P797 (1 mmol.dm^−3^) at different scan rates: 0.1 V.s^−1^ (a), 0.2 V.s^−1^ (b), 0.5 V.s^−1^ (c), 1 V.s^−1^ (d). Inset: variation of the oxidation peak current with the square root of the scan rate.

The cyclic voltammograms of P797 are presented in Figure 2. As shown in Figure 2A, P797 oxidizes along a reversible process that takes place at 0.40 V/SCE, followed by an irreversible peak at 0.85 V/SCE. The reversible oxidation at 0.40 V is ascribed to the Fe(II) → Fe(III) conversion in the ferrocenyl moiety, as already reported for ferrociphenols [33,34,35,36,37,38]. The sluggish irreversible peak at 0.85 V most likely represents the direct oxidation of a phenol group [35].

Focusing on the Fe(II) → Fe(III) reversible oxidation wave, the peak current intensities appear to increase with the square root of the scan rate (inset in Figure 2B), as expected for a diffusion-controlled faradaic wave. Coumarin-functionalized compounds are known to undergo strong adsorption on metal and glassy carbon electrodes [39,40,41], thus deforming the shape of voltammetric signals and even fully passivating the electrode surface. As shown in Figure 2B, the voltammograms remain diffusion-controlled within the scan rates explored, showing that passivation is negligible in our conditions. The diffusion coefficient (D_P**797**_) estimated from peak current intensity—scan rate dependence described by the Randles–Ševčík equation [42] is D_P**797**_ = 2.4 × 10^−6^ ± 0.3 × 10^−6^ cm^2^.s^−1^. 

**Scheme 2:**
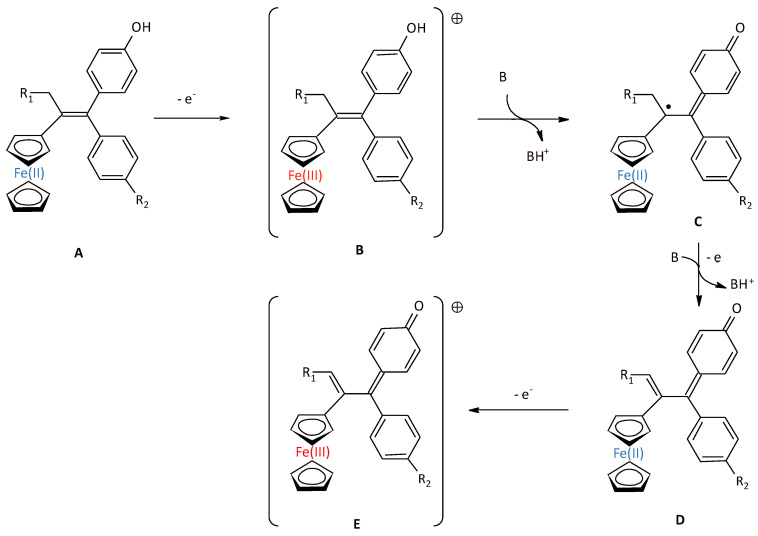
General sequence proposed for the oxidation of ferrociphenols (ferrocifens having at least one phenol substituent) in the presence of a base (adapted from refs. [21,35]). The oxidized Fe(III) states are highlighted in red.

The base-promoted oxidation of ferrociphenol compounds has been explored previously [21] and mostly features the ferrocene-mediated oxidation of the phenol to a quinone methide. In our detailed mechanistic study of ferrociphenols [35], the overall oxidation sequence featured a bielectronic ferrocene-promoted oxidation of ferrociphenol **A** to its quinone methide **D**, which may undergo an additional monoelectronic oxidation to yield **E** (Scheme 2). The electrochemical one-electron oxidation converts **A** into **B**. The addition of a base triggers a second monoelectronic oxidation featuring an intermediate C(sp^3^)-centered radical **C** which undergoes proton coupled electron transfer (PCET) to yield the stable quinone methide **D**. Finally, **D** may undergo an additional 1-electron oxidation to produce **E**. This latter **D** → **E** monoelectronic reversible oxidation wave is overlapped in the irreversible bielectronic wave corresponding to the overall conversion of **A** to **D**.

**Figure 3:**
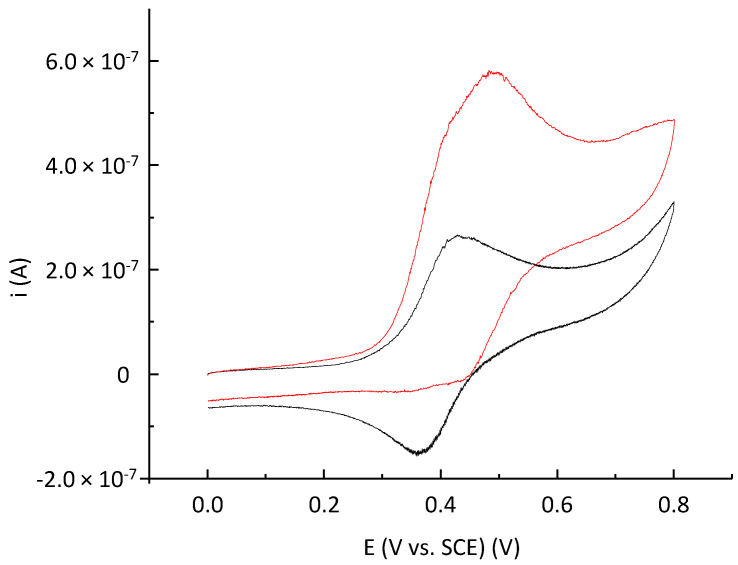
CVs of P797 (1 mmol.dm^−3^) in acetonitrile + TBABF_4_ 0.1 mol.dm^−3^ at a Pt disk electrode (diam 0.5 mm). Scan rate: 0.1 V.s^−1^. Black curve: in the absence of imidazole. Red curve: in the presence of a tenfold molar excess (10 mmol.dm^−3^) of imidazole.

Accordingly, it was interesting to study the electrochemical behavior of the newly prepared coumarin-functionalized ferrociphenol P797 in the presence of a base. Its CVs in the absence and presence of a tenfold excess of imidazole as a base are presented in Figure 3 (red curve). The cyclic voltammograms shown in Figure 3 show the same characteristic evolution in the presence of imidazole as the one described for all the ferrociphenol series [38], suggesting that the base-dependent oxidative sequence depicted in Scheme 2 also applies to P797. As observed in Figure 3, the presence of imidazole triggers a two-electron oxidation sequence at 0.39 V/SCE most likely leading to the formation of a quinone methide intermediate which oxidizes in a reversible wave at 0.49 V/SCE (overlapping the bielectronic wave). Ultimately, the electrochemical oxidation of P797 may involve three electrons (Scheme 2), the third electron most likely accounting for the oxidation of the quinone methide **D** to its ferrocenium analog **E** in Scheme 2, demonstrating that the presence of the coumarin fluorophore does not affect the electrochemical properties of the complex.

In fact, quinone methides (QMs) such as **D** are reactive species and their production in cancer cells has been identified as playing an important role in the cytotoxicity of ferrocifens [43]. Moreover, cancer cells are known to overproduce ROS and RNS [44], making the exploration of the oxidized states biologically relevant. 

### 2.4. Electrochemical Preparation of P797 Oxidized States

**Figure 4:**
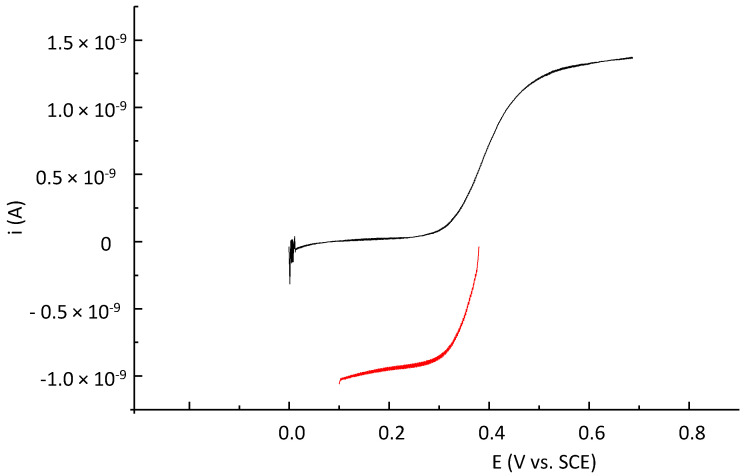
Stationary voltammograms of P797 (0.5 mmol.dm^−3^) in acetonitrile + TBABF_4_ 0.1 mol.dm^−3^ at a 25 µm Pt disk ultramicroelectrode. Scan rate: 0.05 V.s^−1^. Black curve: anodic scan of P797 before electrolysis. Red curve: cathodic scan recorded after a 0.92 Faraday/mole electrolysis at a gold grid anode polarized at 0.7 V/SCE.

We intended to explore the fluorescence properties of the oxidized states of P797. For this purpose, we chose to generate oxidized intermediates from the electrochemical oxidation of P797 in a divided cell, the anodic compartment being fitted with a gold grid electrode. The stepwise electrochemical generation of the successive intermediates met along the oxidative metabolism of ferrociphenols has been described extensively by us [35]. We encourage the reader to obtain further information on the electrolyses in that reference as well as in Section 3.2. The same apparatus, cells and procedures are used in this work to generate the oxidized states of P797. The reversibility of the monoelectronic oxidation observed in Figure 2B allows the quantitative conversion of P797 to its ferrocenium analog (**A** → **B** in Scheme 2). Stationary voltammograms have been recorded at a 25 µm diameter Pt ultramicroelectrode before and after 1 Faraday/mole oxidation of P797 in order to ensure that P797 has been converted to its oxidized Fe(III). A typical evolution of the stationary voltammograms is presented in Figure 4.

Since the cathodic red scan in Figure 4 is recorded from an initial potential value corresponding to the open circuit potential (OCP, i.e., the zero-current Nernstian equilibrium potential of the solution obtained after the 1 Faraday/mol oxidation of P797), one can observe an abrupt intensity change accounting for the strictly cathodic wave displayed. The comparable current intensities of the anodic (black curve, before electrolysis) and cathodic (red curve, after electrolysis) scans in Figure 4 reflect the reversible nature of the oxidation wave observed in the CVs in Figure 2. However, the cathodic plateau current continuously decreases with time over 30 min, revealing an instability of the Fe(III) redox state in our conditions. Accordingly, the electrochemical divided cell was placed in ice for preparative electrolysis in order to slow down the degradation of the electrogenerated Fe(III) states.

### 2.5. Fluorescence Properties of P797 in Its Reduced and Oxidized Forms

**Figure 5:**
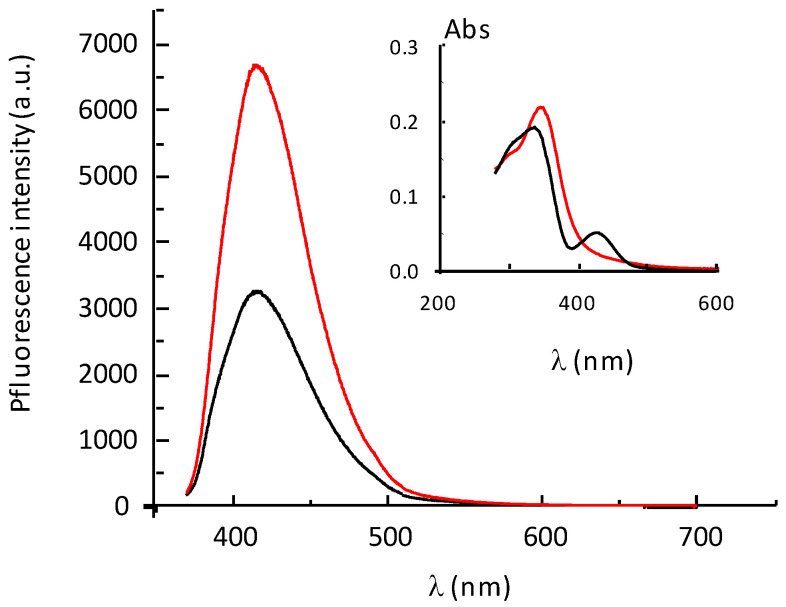
Uv-vis absorption (inset) and fluorescence emission spectra of P797 at 10^−5^ mol.dm^−3^ in acetonitrile + TBABF_4_ 0.1 mol.dm^−3^. Black curve: before electrolysis (Species **A** in Scheme 2). Red curve: after a 1 Faraday/mole oxidation at 0.7 V/SCE (Species **B** in Scheme 2). Excitation wavelength λ_ex_ = 350 nm for the fluorescence spectra.

P797 in its oxidized state (Fe(III)) was electrogenerated as described above and its fluorescence properties were compared to those in its reduced state. The solution of oxidized compound was analyzed within 15 min after its preparation, in order to minimize degradation. The fluorescence and UV-vis spectra of reduced and oxidized P797 are presented in Figure 5.

As shown in Figure 5, oxidized P797 (species **B**, red curve) exhibits (i) slightly shifted absorption spectrum (from 337 nm for P797 to 350 nm for its oxidized form) and (ii) a two-fold enhancement of the fluorescence intensity compared to the original reduced compound. This difference between the reduced and oxidized species **A** and **B** is in line with the occurrence of a photo-induced electron transfer (PET) between the coumarin fluorophore and the metal center in **A**, that should result in fluorescence quenching. However, PET is only partial since species **A** is fluorescent upon excitation at 350 nm. Conversely, the PET mechanism no longer applies for the oxidized intermediate **B**, resulting in higher fluorescence. 

**Figure 6:**
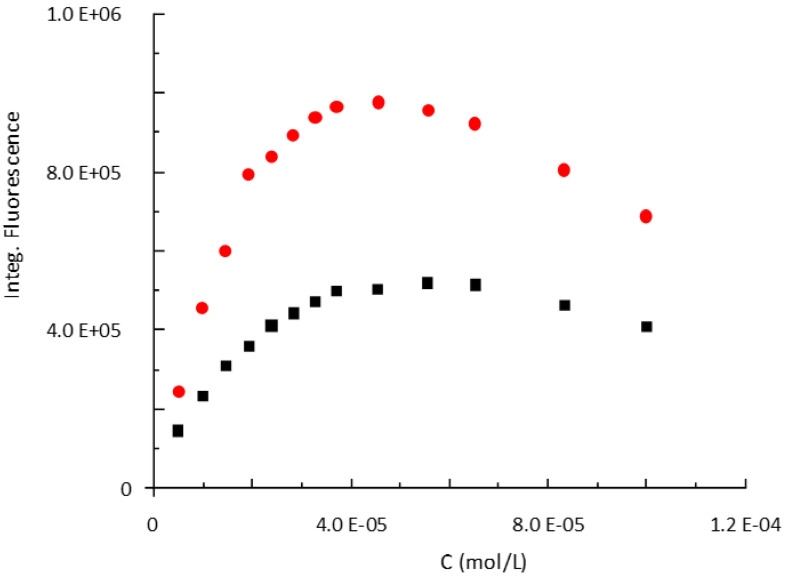
Integrated fluorescence intensity of P797 in its reduced (black marks, **A**) and oxidized (red marks, **B**) forms as a function of its concentration in acetonitrile+ TBABF_4_ 0.1 mol.dm^−3^.

We have then checked whether fluorescence self-quenching could take place on both redox states of P797. The corresponding fluorescence intensity–concentration dependence is presented in Figure 6.

Self-quenching of P797 in its reduced form (black marks) becomes significant at concentrations higher than 15 µmol.dm^−3^, with a maximum fluorescence intensity at ca. 50 µmol.dm^−3^ (Figure 6). Its oxidized derivative (red marks) exhibits a similar dependence with concentration, although its fluorescence yield remains higher. Accordingly, we have recorded the fluorescence spectra of P797 at 10 µmol.dm^−3^ in order to minimize its self-quenching.

### 2.6. Determination of the Quantum Yields of P797 in Its Reduced and Oxidized Form

We have then undertaken an estimation of the quantum yields of the reduced and oxidized forms of P797 by comparing their fluorescence emission with a known standard (9,10-diphenylanthracene; DPA) that exhibits fluorescence upon excitation at the same wavelength ($\Phi_{X}$_exc_ = 350 nm). The quantum yield was estimated from the emission/absorption gradients obtained from the absorbance at 350 nm and fluorescence intensity (integrated over the full spectrum) [45,46,47]. Using the same spectrophotometers and fluorimeters for the sample and the standard, the quantum yield $\Phi_{X}$_x_ of fluorescence of a given compound x is given by equation the following Equation (1):

(1) $$\Phi_{X}={\;\Phi }_{\mathrm{std}}\;\frac{\mathrm{Grad}\left(x\right)}{\mathrm{Grad}\left(\mathrm{std}\right)}\;{\left(\frac{\eta_{x}}{\eta_{\mathrm{std}}}\right)}^{2}\;$$

Equation (1). Gradient(x) [Grad(x)] and gradient standard [Grad(std)] are the slopes of the curve plotting integrated fluorescence against absorbance at λ_ex_ of compound x under study or the standard, respectively. Values η_x_ and η_std_ are the refractive indexes of the solvents used for the compound and the standard (Φ_std_ = 1.00 in our conditions [40]. 

**Figure 7:**
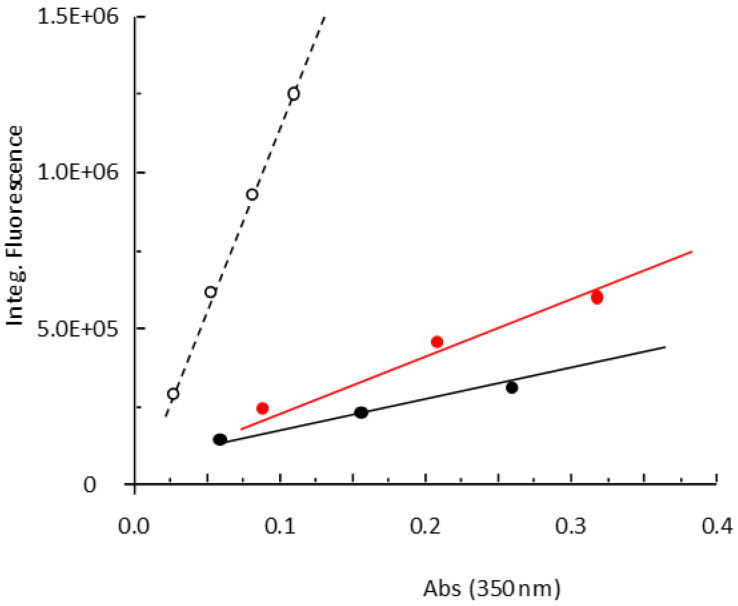
Integrated fluorescence vs. absorbance gradients of DPA (reference: white circles and dotted line), P797 (black circles and black line) and oxidized P797 solutions-1 Faraday/mole electrooxidation of P797 (red circles and red line) in acetonitrile + TBABF_4_ 0.1 mol.dm^−3^. Concentrations are in the range 10^−5^–4 × 10^−5^ mol.dm^−3^.

The values of Grad(x) and Grad (std) associated with P797 are the slopes of the curves presented in Figure 7.

From the linear variations observed in Figure 7, the fluorescence quantum yield of the reduced and oxidized states of P797 can be estimated to Φ_P797_ = 0.06 (reduced state) and Φ_P797+_ = 0.135 (oxidized state). The value of the Φ_P797_/Φ_P797+_ ratio is consistent with the fluorescence intensity differences observed in Figure 4 and Figure 6.

### 2.7. Fluorescence Properties of P797 in the Presence of Imidazole 

**Figure 8:**
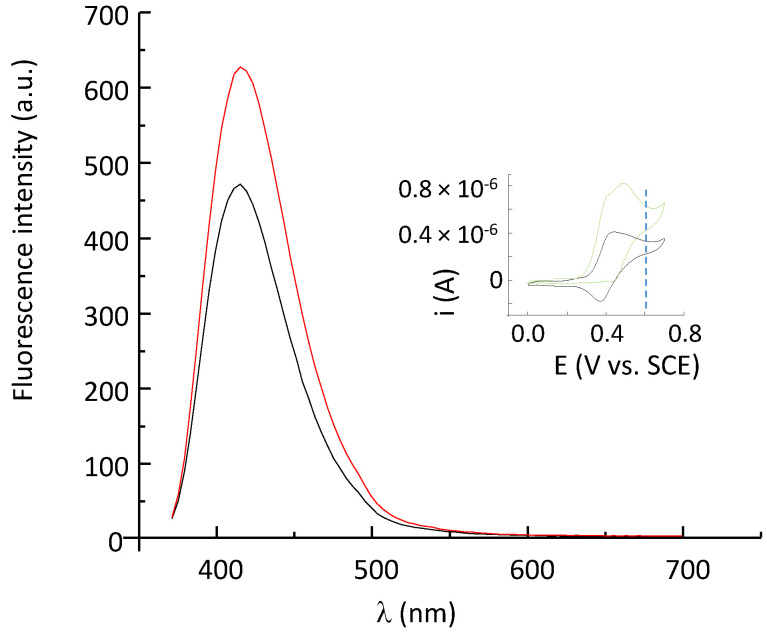
Fluorescence emission spectra of a 1:10 mixture of P797 and imidazole before and after electrolysis ([P797] = 5.10^−6^ mol.dm^−3^, [Imidazole] = 5.10^−5^ mol.dm^−3^ in acetonitrile + TBABF_4_ 0.1 mol.dm^−3^). Black curve: before electrolysis (Species **A** in Scheme 2). Red curve: after a 3 Faraday/mole oxidation at 0.6 V/SCE (Species **E** in Scheme 2). Inset: CVs recorded before electrolysis. Black curve: no imidazole added. Green curve: addition of a tenfold excess of imidazole (the vertical blue dashed line shows the potential applied for the electrolysis): Excitation wavelength λ_ex_ = 350 nm.

We next investigated the fluorescence properties of P797 electrolyzed in the presence of imidazole (as a base). The corresponding spectra are presented in Figure 8.

The oxidation of P797 in the presence of imidazole engages three electrons, as verified from the integration of the current with time (0.7 C passed at 0.6 V vs. SCE for a theoretical charge of 0.72 C for a three-electron process). Since the duration of the electrolysis (900 s) is roughly a hundred times that of the time window required to record a cyclic voltammogram at 0.1 V.s^−1^, one can expect the three-electron oxidation of P797 to be complete under electrolysis conditions. Note that intermediate **C** is instable (in other words **C** is a transient species) in the presence of both imidazole and oxidizing conditions. As a result, the major species present after electrolysis should be the ferrocenium cation **E**, obtained along the full three-electron oxidation sequence shown in Scheme 2. The fluorescence spectra in Figure 8 may therefore be assigned, respectively, to precursor **A** (black curve, before electrolysis) and product **E** (red curve, after electrolysis) as the major fluorescent species. Here, again, the oxidized Fe(III) product **E** (a quinone methide) exhibits an increased fluorescence emission upon excitation at 350 nm compared to the original Fe(II) precursor P797. The PET invoked to explain the higher fluorescence emission of the oxidized state of P797 (ferrocenium cation B—Figure 4 and Figure 5) should also take place in the redox couple **D**/**E** of its quinone methide.

**Figure 9:**
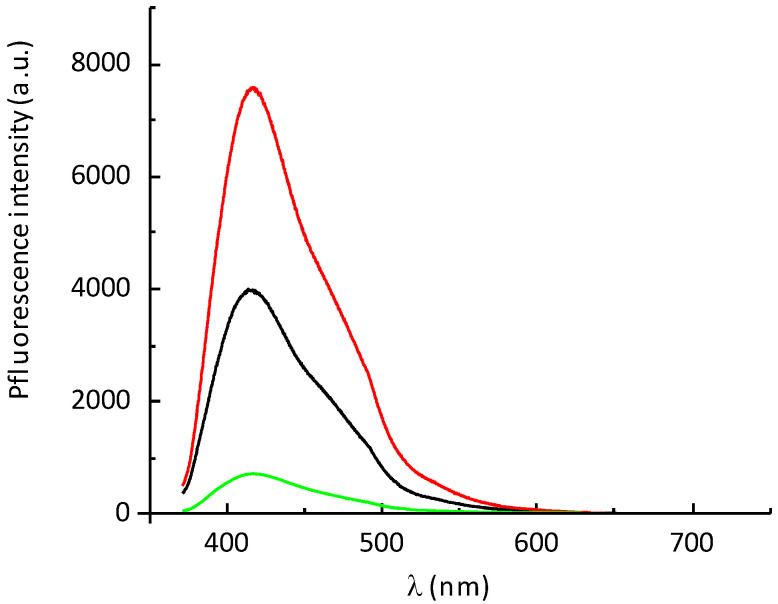
Fluorescence emission spectra of P797 at 5 × 10^−6^ mol.dm^−3^ in acetonitrile + TBABF_4_ 0.1 mol.dm^−3^. [Imidazole] = 5 × 10^−5^ mol.dm^−3^. Black curve: before electrolysis (Species **A** in Scheme 2). Red curve after 1 Faraday/mole oxidation at 0.7 V/SCE (Species **B** in Scheme 2). Green curve: after addition of a tenfold excess of imidazole as a base on the previous (red curve) solution (Species **C** in Scheme 2). Excitation wavelength λ_ex_ = 350 nm.

We eventually carried out an additional experiment where imidazole was added to the electrochemically generated one-electron oxidation product of P797 (intermediate B in Scheme 2). In fact, if B converts to radical **C** upon addition of a base, the fluorescence emission should be affected, considering that intermediate **C** is formed along the base-triggered intramolecular electron transfer between the phenol and the ferrocenium Fe(III) “antenna”. The formation of **C** is likely considering that the addition of imidazole on B is not accompanied by an electrolysis, so that only chemical evolution is possible from the B + imidazole solution. The corresponding fluorescence spectra are shown in Figure 9.

One can observe in Figure 9 that the one-electron oxidation of P797 is accompanied by an increase in fluorescence, as already commented in Figure 5. The addition of imidazole to the solution of oxidized compound, i.e., on intermediate B in Scheme 2, results in a dramatic decrease in fluorescence intensity (red to green trace). This fluorescence quenching completely agrees with the formation of a formal Fe(II) redox state, depicted as intermediate **C** in the oxidation sequence. On the one hand, monitoring fluorescence along the oxidation sequence strengthens the mechanism established previously from stepwise electrochemical and electron spin resonance experiments [35]. On the other hand, the large differences in fluorescence intensity observed in Figure 9 between P797 (precursor **A**, black curve) and intermediate **C** (green curve) may account for a radical-centered fluorescence quenching mechanism for intermediate **C**, as quenching of fluorescence by radicals has long been known [48]. This brings another brick to the oxidative mechanistic scheme of coumarin-functionalized ferrocifens.

## 3. Materials and Methods

### 3.1. Synthesis of Coumarin-Functionalized Ferrociphenols P794 and P797

All ^1^H and ^13^C-NMR spectra were acquired on Bruker 300 and 400 MHz spectrometers, in acetone-d^6^ as the solvent. The ^1^H and ^13^C NMR spectra of **2a** and **2b** intermediates and of the final **3a** and **3b** compounds (i.e P794 and P797) are provided in the supplementary Material. High resolution mass spectra (HRMS) were performed at the MS3 platform of Sorbonne Université. Thin layer chromatography was performed on silica gel 60 GF254. Purification by column chromatography was performed on the Puriflash 430 system (Interchim) using pre-packed silica gel cartridges (Grace). Ketones **1a** (*n* = 3) and **1b** (*n* = 4) were synthesized from ferrocene and 5-hexynoic acid or 6-heptynoic acid, respectively, as described by Plazuk and Zakrzewski [49]. 3-azido-7-hydroxycoumarin **4** was prepared according to a published procedure [50]. Other reagents were obtained from commercial suppliers and used as received.

#### 3.1.1. Synthesis of **2a** and **2b** via a McMurry Coupling

Titanium chloride was added dropwise to a suspension of zinc powder in dry THF at 10–20 °C. The mixture was heated at reflux for 2 h. A second solution was prepared by dissolving the two ketones in dry THF. This latter solution was added dropwise to the first solution and then the reflux was continued for 2 h. After cooling to room temperature, the mixture was stirred with water and dichloromethane. The mixture was acidified with dilute hydrochloric acid until the dark color disappeared and was then decanted. The aqueous layer was extracted with dichloromethane and the combination of organic layers was dried on magnesium sulfate. After concentration under reduced pressure, the crude product was flash chromatographed on silica gel column with an ethyl acetate/cyclohexane (1:2) mixture as eluent to afford the alkynes.

#### 2-Ferrocenyl-1,1-bis-(4-hydroxyphenyl)-hept-1-en-6-yne (**2a**)

2a was prepared using 1a (2.345 g, 8.37 mmol), 4,4′-dihydroxybenzophenone (3.586 g, 16.7 mmol), zinc (3.283 g, 50.2 mmol), titanium(IV) chloride (6.352 g, 3.68 mL, 33.5 mmol) to afford 2a as an orange solid with a yield 63%. ^1^H NMR (acetone-d^6^): δ 1.60–1.75 (m, 2H, CH_2_), 2.02–2.14 (m, 2H, CH_2_), 2.35 (t, J = 2.6 Hz, 1H, alkyne), 2.72–2.83 (m, 2H, CH_2_), 3.99 (t, J = 1.9 Hz, 2H, C_5_H_4_), 4.08 (t, J = 1.9 Hz, 2H, C_5_H_4_), 4.14 (s, 5H, Cp), 6.71 (d, J = 8.7 Hz, 2H, C_6_H_4_), 6.82 (d, J = 8.7 Hz, 2H, C_6_H_4_), 6.88 (d, J = 8.7 Hz, 2H, C_6_H_4_), 7.07 (d, J = 8.7 Hz, 2H, C_6_H_4_), 8.22 (s, 1H, OH), 8.25 (s, 1H, OH). ^13^C NMR (acetone-d^6^): δ 19.0 (CH_2_), 29.8 (CH_2_), 34.8 (CH_2_), 68.6 (2CH C_5_H_4_), 69.8 (5CH Cp), 70.0 (2CH C_5_H_4_), 70.1 (C alkyne), 85.0 (CH alkyne), 88.5 (C C_5_H_4_), 115.8 (2CH C_6_H_4_), 115.9 (2CH C_6_H_4_), 131.3 (2CH C_6_H_4_), 131.8 (2CH C_6_H_4_), 134.5 (C), 137.0 (C), 137.4 (C), 139.7 (C), 156.6 (C), 156.7 (C). IR (KBr, ν cm^−1^): 3408 (OH), 2259 (triple bond). MS (CI, NH_3_) *m*/*z*: 463 [M + H]^+^. HRMS (ESI, C_29_H_26_FeO_2_: [M]^+^.) calcd: 462.12767, found: 462.12763.

#### 2-Ferrocenyl-1,1-bis-(4-hydroxyphenyl)-oct-1-en-7-yne (**2b**)

2b was prepared using 1b (5.3 g, 18.02 mmol), 4,4′-dihydroxybenzophenone (3.86 g, 18 mmol), zinc (7.067 g, 108.1 mmol), titanium(IV) chloride (13.672 g, 7.92 mL, 72.1 mmol) to afford 2b as an orange solid with a yield 27%. ^1^H NMR (acetone-d^6^): δ 1.36–1.50 (m, 2H, CH_2_), 1.53–1.70 (m, 4H, CH_2_), 2.27 (t, J = 2.7 Hz, 1H, CH alkyne), 2.59–2.71 (m, 2H, CH_2_), 3.94 (t, J = 1.9 Hz, 2H, C_5_H_4_), 4.06 (t, J = 1.9 Hz, 2H, C_5_H_4_), 4.12 (s, 5H, Cp), 6.70 (d, J = 8.6 Hz, 2H, C**_6_**H_4_), 6.81 (d, J = 8.6 Hz, 2H, C**_6_**H_4_), 6.87 (d, J = 8.6 Hz, 2H, C**_6_**H_4_), 7.06 (d, J = 8.6 Hz, 2H, C**_6_**H_4_), 8.16 (s, 1H, OH), 8.20 (s, 1H, OH). ^13^C NMR (acetone-d^6^): δ 18.5 (CH_2_), 29.5 (CH_2_), 30.2 (CH_2_), 34.9 (CH_2_), 68.6 (2CH C_5_H_4_), 69.9 (5CH Cp), 70.1 (2CH C_5_H_4_ + C alkyne), 85.0 (CH alkyne), 88.5 (C C_5_H_4_), 115.8 (2CH C_6_H_4_), 115.9 (2CH C_6_H_4_), 131.3 (2CH C_6_H_4_), 131.8 (2CH C_6_H_4_), 135.1 (C), 137.2 (C), 137.5 (C), 139.4 (C), 156.6 (C), 156.7 (C).

#### 3.1.2. Synthesis of Ferrocifen Coumarin Dyads **3a** and **3b** via a Click Coupling Procedure

2a or 2b (0.54 mmol) was dissolved in ethanol (8 mL) then diluted with H_2_O (6 mL). 3-azido-7-hydroxycoumarin 4, CuSO_4_.5H_2_O (0.068 g in 1 mL H_2_O, 0.27 mmol) and sodium ascorbate (0.107 g in 1 mL H_2_O, 0.54 mmol) were added and the reaction mixture was stirred at room temperature in the dark for 18 h. EtOAc (50 mL) and H_2_O (40 mL) were added and the aqueous solution was extracted with EtOAc (3 × 50 mL). The organic layers were combined and dried over Na_2_SO_4_. After concentration under reduced pressure, the crude product was flash chromatographed on silica gel column with a ethyl acetate/cyclohexane (1:2) mixture as eluent to afford the coumarin derivatives **3a,b**.

#### 7-Hydroxy-3-(4-[35]-[1,2,3]triazol-1-yl)-chromen-2-one (**3a**, P794) (0.24 g, 81% Yield, Orange Solid)

^1^H NMR (acetone-d^6^): δ 1.85–2.00 (m, 2H, CH_2_), 2.66–2.78 (m, 4H, CH_2_), 3.93 (t, J = 1.9 Hz, 2H, C_5_H_4_), 4.04 (t, J = 1.9 Hz, 2H, C_5_H_4_), 4.09 (s, 5H, Cp), 6.70 (d, J = 8.4 Hz, 2H, C_6_H_4_), 6.78 (d, J = 8.4 Hz, 2H, C_6_H_4_), 6.87 (d, J = 8.4 Hz, 2H, C_6_H_4_), 2.86–2.93 (m, 1H, coumarin), 6.95–7.04 (m, 1H, coumarin), 7.04 (d, J = 8.4 Hz, 2H, C_6_H_4_), 7.77 (d, J = 8.1 Hz, 1H, coumarin), 8.08 (s, 1H, aromatic), 8.16–8.94 (s broad, 2H, OH), 8.49 (s, 1H, aromatic). ^13^C NMR (acetone-d^6^): δ 26.2 (CH_2_), 31.2 (CH_2_), 35.0 (CH_2_), 68.6 (2CH C_5_H_4_), 69.9 (5CH Cp), 70.0 (2CH C_5_H_4_), 88.5 (C C_5_H_4_), 103.3 (CH), 112.1 (C), 115.0 (CH), 115.8 (2CH C_6_H_4_), 115.9 (2CH C_6_H_4_), 121.3 (C), 123.0 (CH), 131.3 (2CH C_6_H_4_), 131.6 (CH), 131.8 (2CH C_6_H_4_), 135.0 (C), 135.4 (CH), 137.1 (C), 137.4 (C), 139.5 (C), 148.3 (C), 155.9 (C), 156.6 (C), 156.7 (C), 157.1 (C), 162.9 (CO). HRMS (ESI, C_38_H_31_FeN_3_O_5_: [M]^+^.) calcd: 665.16076, found: 665.16092.

#### 7-Hydroxy-3-(4-[15,33,36]-[1,2,3]triazol-1-yl)-chromen-2-one (**3b**, P797). (0.25 g, 68% Yield, Orange Solid)

^1^H NMR (acetone-d^6^): δ 1.53–1.75 (m, 4H, CH_2_-CH_2_), 2.61–2.75 (m, 4H, CH_2_), 3.92 (t, J = 1.9 Hz, 2H, C_5_H_4_), 4.04 (t, J = 1.9 Hz, 2H, C_5_H_4_), 4.11 (s, 5H, Cp), 6.70 (d, J = 8.5 Hz, 2H, C_6_H_4_), 6.80 (d, J = 8.5 Hz, 2H, C_6_H_4_), 6.86 (d, J = 8.5 Hz, 2H, C_6_H_4_), 2.86–2.92 (m, 1H, coumarin), 6.99 (d, J = 8.4 Hz, 1H, coumarin), 7.06 (d, J = 8.5 Hz, 2H, C6H4), 7.76 (d, J = 8.5 Hz, 1H, coumarin), 7.95–8.43 (s broad, 2H, OH), 8.24 (s, 1H, aromatic), 8.48 (s, 1H, aromatic). ^13^C NMR (acetone-d^6^): δ 25.9 (CH_2_), 30.2 (CH_2_), 30.6 (CH_2_), 35.2 (CH_2_), 68.6 (2CH C_5_H_4_), 69.9 (5CH Cp), 70.1 (2CH C_5_H_4_), 88.5 (C C_5_H_4_), 103.3 (CH), 112.1 (C), 115.1 (CH), 115.8 (2CH C_6_H_4_), 115.9 (2CH C_6_H_4_), 121.3 (C), 122.9 (CH), 131.3 (2CH C_6_H_4_), 131.6 (CH), 131.8 (2CH C6H4), 135.2 (CH + C), 137.2 (C), 137.5 (C), 139.3 (C), 148.5 (C), 155.8 (C), 156.6 (C), 156.7 (C), 157.1 (C), 162.9 (CO). HRMS (ESI, C_39_H_33_FeN_3_O_5_: [M]^+^.) calcd: 679.17641, found: 679.17650.

### 3.2. Electrochemical and Fluorescence Studies

Cyclic voltammetry and electrolyses were performed in acetonitrile where tetrabutylammonium tetrafluoroborate (TBABF_4_) 0.1 M was introduced as supporting electrolyte. An Autolab PGSTAT 20 potentiostat (Metrohm, Switzerland) was used with a three-electrode electrochemical cell, cyclic voltammograms were recorded at a 0.5 mm home-made Pt working electrode (Goodfellow). Saturated calomel electrode (SCE, Radiometer) was used as reference electrode, and a platinum wire (Goodfellow) as counter electrode. The reference electrode was separated from the bulk solution by a fritted-glass bridge filled with the solution of supporting electrolyte (0.1 M TBABF_4_ in MeCN). CV experiments were recorded after at least 10 min of argon purging, at room temperature. 

Electrolyses were performed in a divided 2 × 5 mL cell where the compartments are separated by a porosity 4 sintered glass. P797 was introduced in the anodic compartment with the same electrolyte (MeCN with 0.1 M TBABF_4_), see reference [32] for further details regarding the electrochemical cells and setup. Aliquots (20 µL) of the solutions introduced or electrolyzed in the anodic compartment were transferred to a quartz UV-Vis 2 mL cuvette to record the absorption and fluorescence spectra. Perkin Elmer Lambda 45 spectrometer was used in absorption measurements and JASCO FP-8300 fluorometer in fluorescence measurements, from 2 mL solutions placed in quartz cuvettes.

## 4. Conclusions

Finally, the fluorescence emission of the model coumarin-functionalized ferrociphenol P797 appears to strongly depend on the oxidation state of its ferrocene group. The higher fluorescence yield exhibited by the Fe(III) intermediates suggests the occurrence of a fluorescence quenching mechanism on its reduced Fe(II) analogs, most likely through photoinduced electron transfer from Fe(II) to the coumarin. We have shown that a carbon-centered radical intermediate in the oxidation sequence also enables a quenching of fluorescence. Under oxidative stress, a cancer cell overproducing reactive oxygen or nitrogen species would provide both the oxidizing and pH-buffered environment prone to convert a molecule such as P797 to its three-electron oxidized ferrocenium quinone methide. The resulting fluorescence is expected to increase and this property opens avenues in the imaging of such ferrociphenols and more generally on ferrocene-functionalized coumarins in cell cultures.

## Supplementary Materials

The following supporting information can be downloaded at: https://www.mdpi.com/article/10.3390/molecules27196690/s1, The ^1^H and ^13^C NMR spectra of **2a**, **2b**, **3a** and **3b**: Figure S1: ^1^H and ^13^C NMR spectra of **2a**; Figure S2: ^1^H and ^13^C NMR spectra of **2b**; Figure S3: ^1^H and ^13^C NMR spectra of **3a**; Figure S4: ^1^H and ^13^C NMR spectra of **3b**.

## Sample Availability

Samples of the compounds are available from Anne Vessières and Michèle Salmain.
