# Supplementary material for: Synthesis, Electrochemical and Fluorescence Properties of the First Fluorescent Member of the Ferrocifen Family and of Its Oxidized Derivatives
Source: Molecules. 2022 Oct 8;27(19):6690. doi: 10.3390/molecules27196690 (PMC9571219; doi:10.3390/molecules27196690)

# Supplementary Materials

# Synthesis, Electrochemical and Fluorescence Properties of the First Fluorescent Member of the Ferrocifen Family and its Oxidized Derivatives.

Charles Fayolle, Pascal Pigeon, Nathalie Fischer-Durand, Michèle Salmain, Olivier Buriez, Anne Vessières, Eric Labbé

**Table of contents**

Figure S1. Structural data for compound **2a**
Figure S2. Structural data for compound **2b**
Figure S3. Structural data for compound **3a** (**P794**)
Figure S4. Structural data for compound **3b** (**P797**)

Figure S1. $^{1}H$ and $^{13}C$ NMR spectra of **2a**

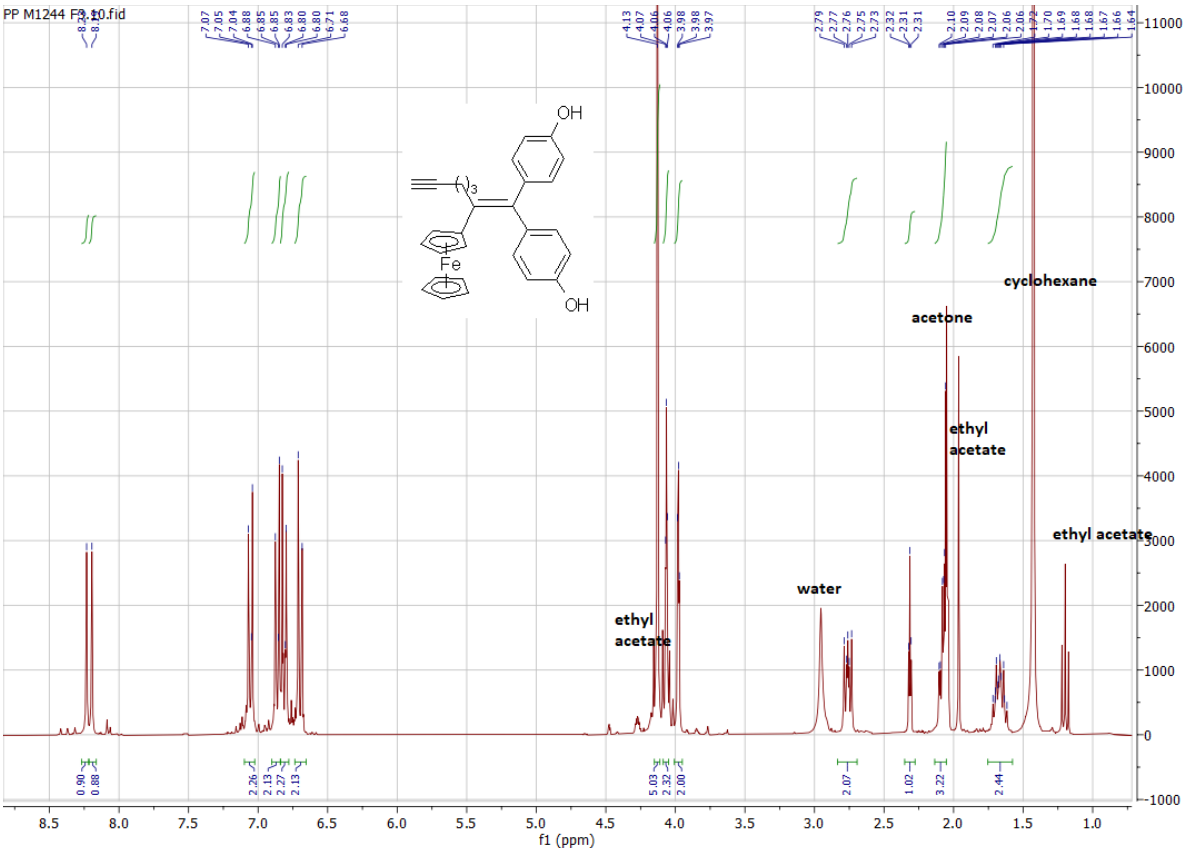


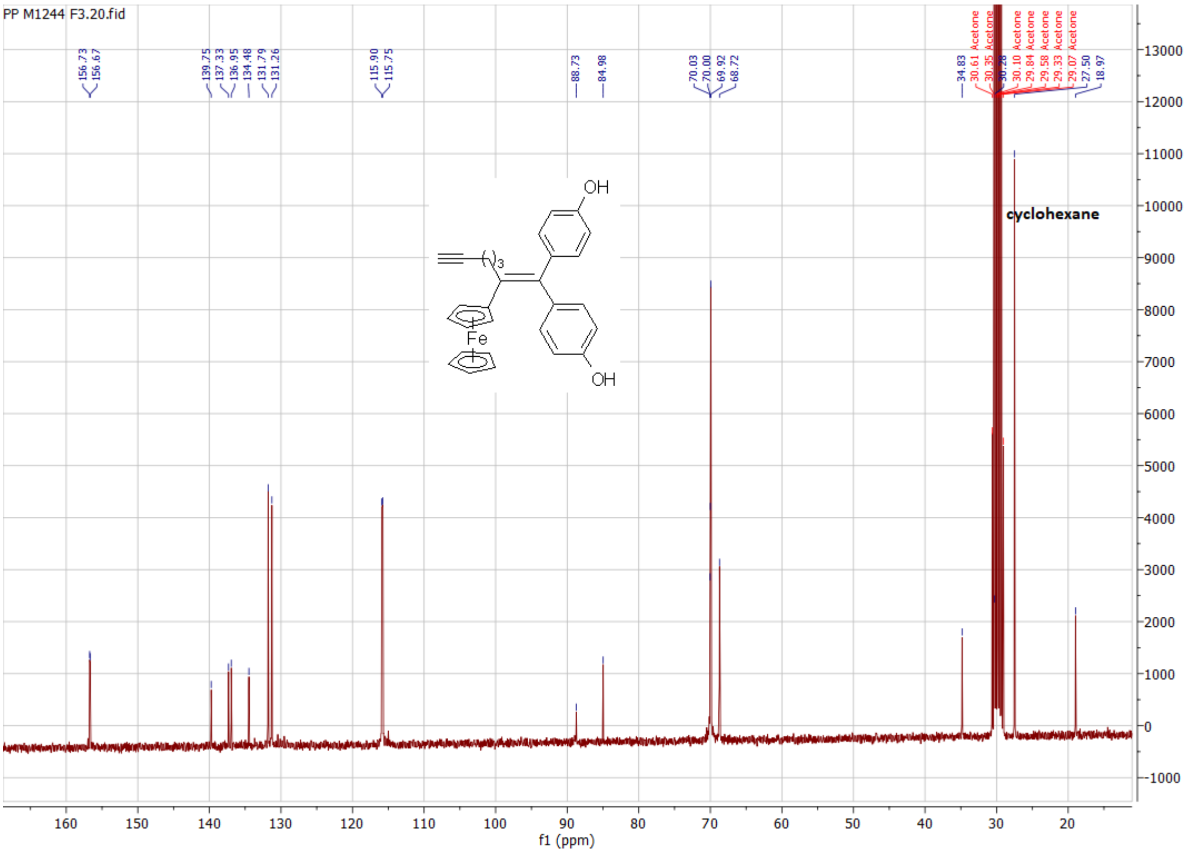

Figure S2. $^{1}H$ and $^{13}C$ NMR spectra of **2b**

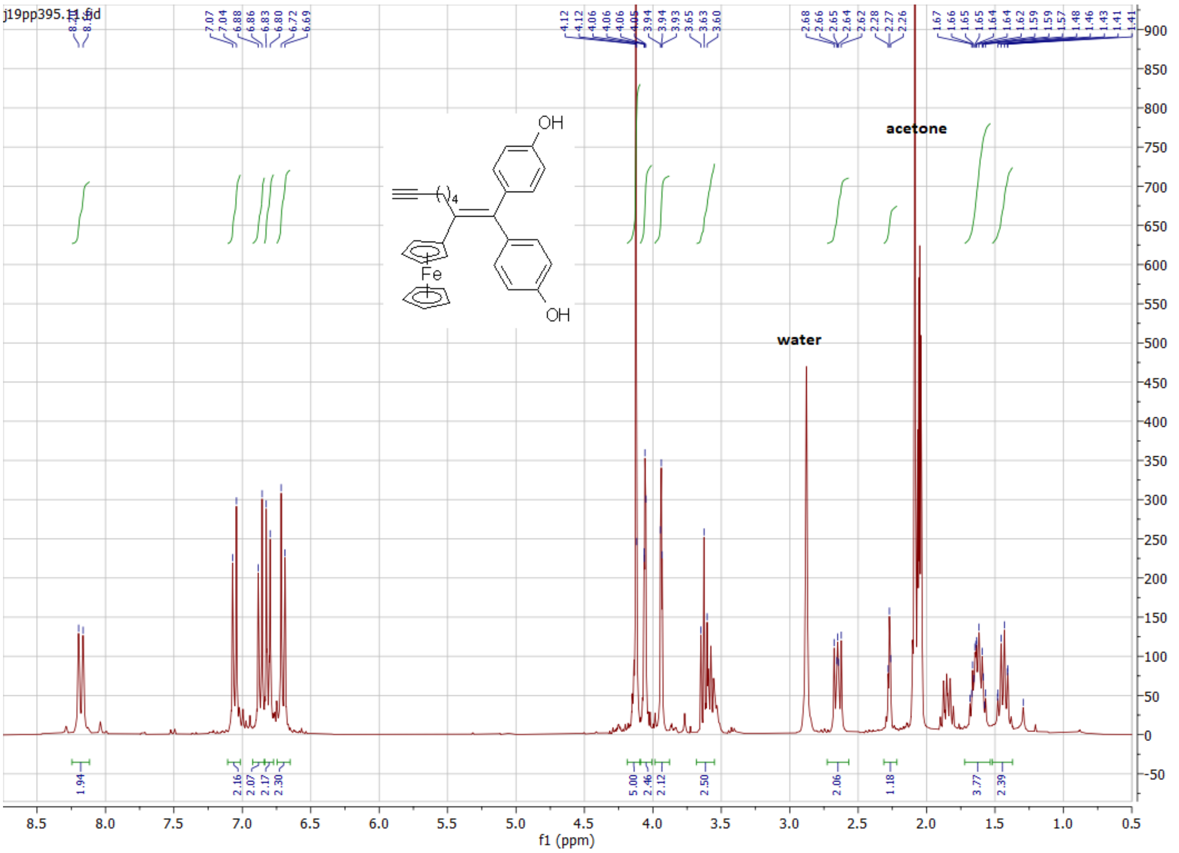


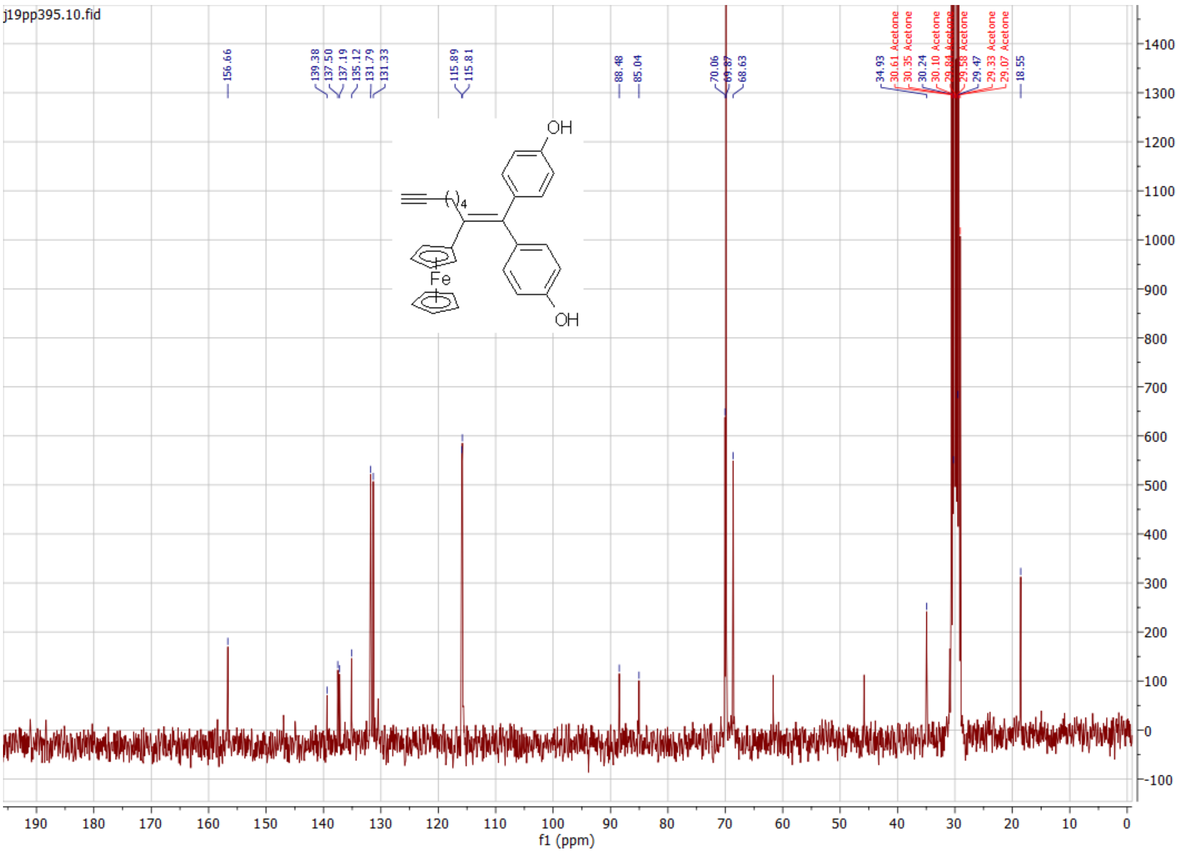

Figure S3. $^{1}$H and $^{13}$C NMR spectra of **3a (P794)**

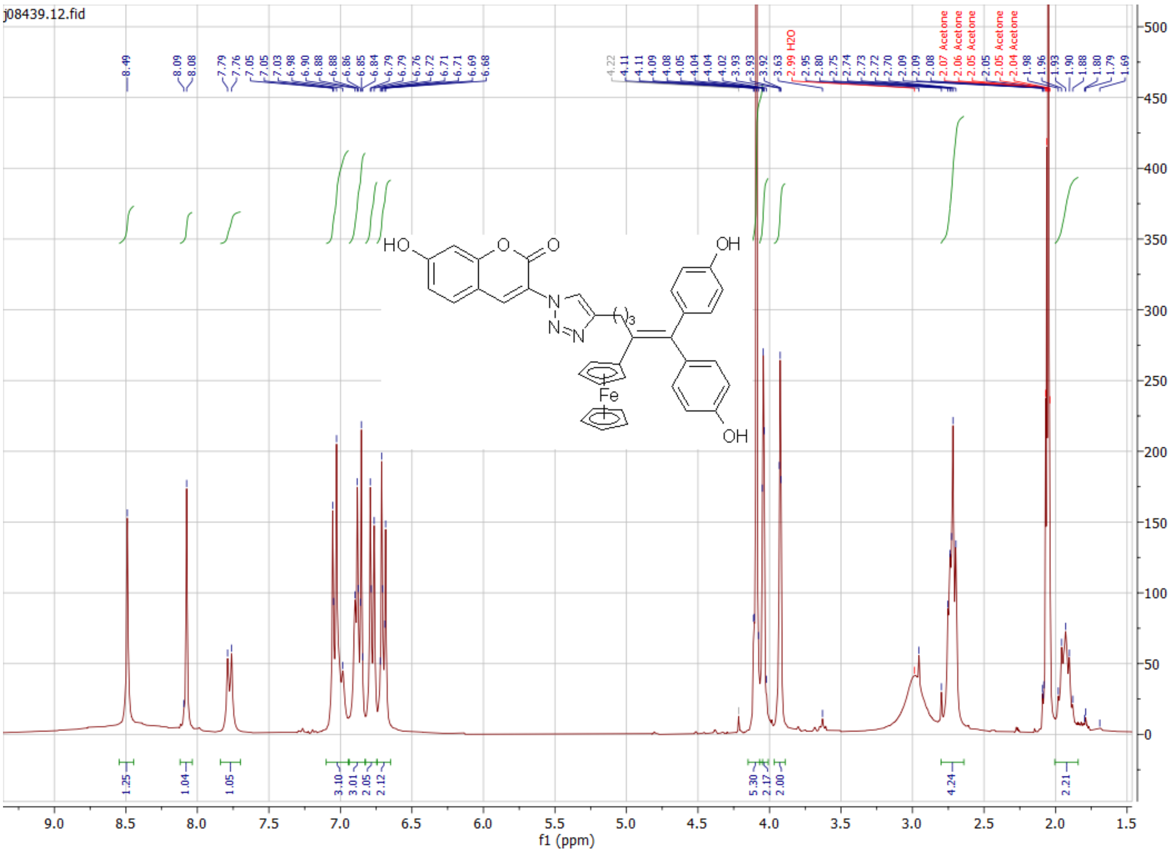


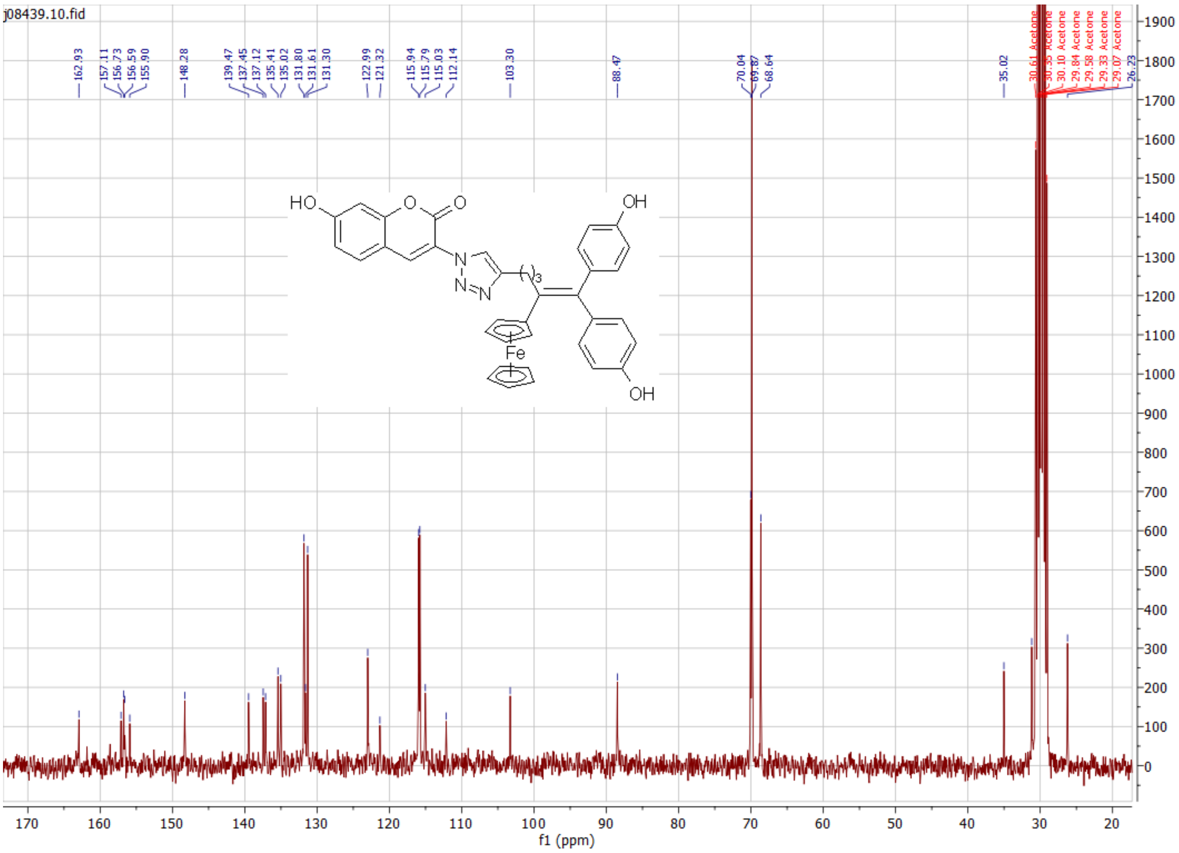

Figure S4. $^{1}H$ and $^{13}C$ NMR spectra of **3b** (**P797**)

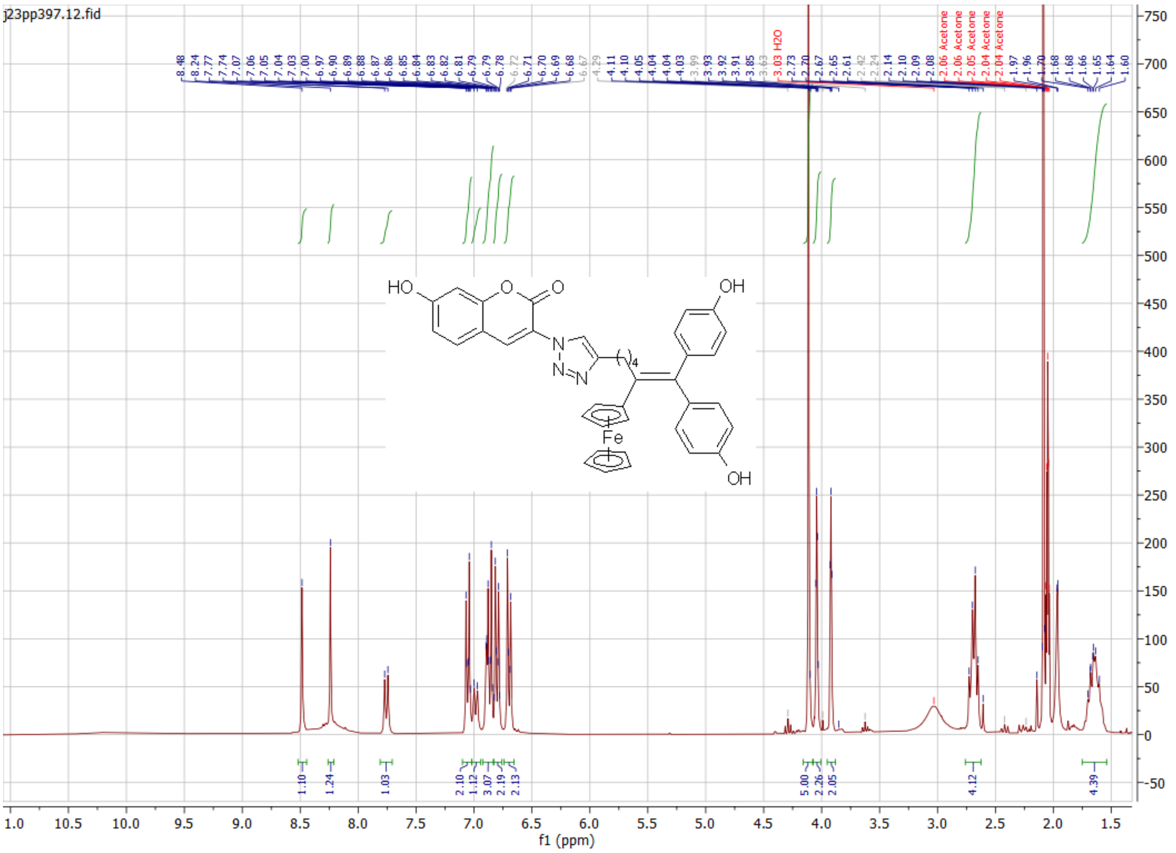


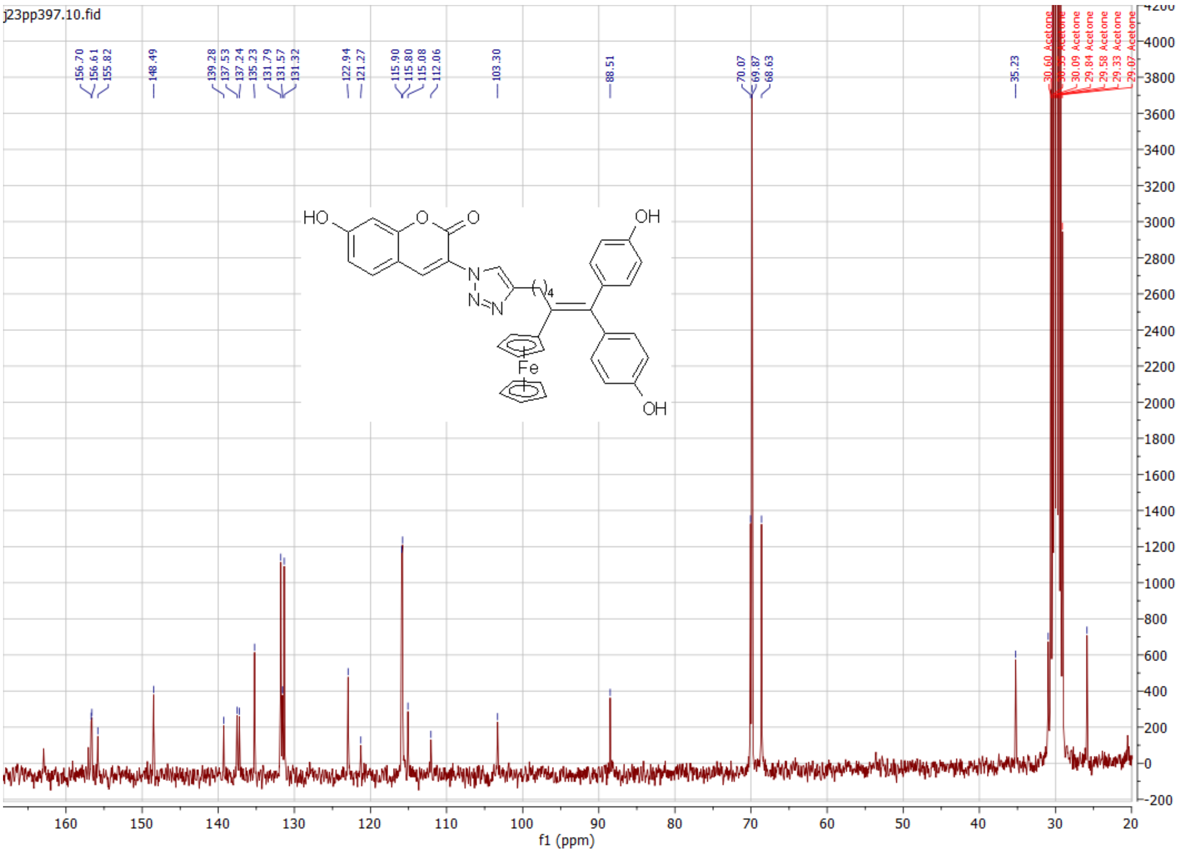

Supplement: Supplementary file 1 [file molecules-27-06690-s001.zip › molecules-1941130-supplementary.pdf]
